# Prevalence of SARS-CoV-2 Antibodies after First 6 Months of COVID-19 Pandemic, Portugal

**DOI:** 10.3201/eid2711.210636

**Published:** 2021-11

**Authors:** Luísa Canto e Castro, Ana Helena Guia Pereira, Rita Ribeiro, Catarina Alves, Luís Veloso, Vera Vicente, Dalila Alves, Inês Domingues, Cláudia Silva, Andreia Gomes, Marta Serrano, Ângela Afonso, Marc Veldhoen, Maria José Rego de Sousa, José Germano Rego de Sousa, Germano de Sousa, Maria M. Mota, Bruno Silva-Santos, Ruy M. Ribeiro

**Affiliations:** Fundação Francisco Manuel dos Santos, Lisbon, Portugal (L. Canto e Castro);; Universidade de Lisboa, Lisbon (L. Canto e Castro, I. Domingues, C. Silva, A. Gomes, M. Serrano, Â. Afonso, M. Veldhoen, M.M. Mota, B. Silva-Santos, R.M. Ribeiro);; Centro de Medicina Laboratorial Germano de Sousa, Lisbon (A.H.G. Pereira, R. Ribeiro, M.J. Rego de Sousa, J.G. Rego de Sousa, G. de Sousa);; CTI Clinical Trial and Consulting Services, Portugal (C. Alves, L. Veloso, V. Vicente, D. Alves);; Los Alamos National Laboratory, Los Alamos, New Mexico, USA (R.M. Ribeiro)

**Keywords:** coronavirus disease, COVID-19, pandemic, severe acute respiratory syndrome coronavirus 2, SARS-CoV-2, coronaviruses, viruses, respiratory infections, population prevalence, seroepidemiology, antibodies, zoonoses, Portugal

## Abstract

In September 2020, we tested 13,398 persons in Portugal for antibodies against severe acute respiratory syndrome coronavirus 2 by using a quota sample stratified by age and population density. We found a seroprevalence of 2.2%, 3–4 times larger than the official number of cases at the end of the first wave of the pandemic.

Severe acute respiratory syndrome coronavirus 2 (SARS-CoV-2) has spread rapidly worldwide during 2020–2021, but incidence has been highly variable in different countries and is difficult to estimate. In Portugal, which has ≈10.3 million inhabitants, the burden of disease, cases, and deaths was similar to or less than that for neighboring countries during the first wave of the coronavirus disease (COVID-19) pandemic, through September 2020 (Appendix Figure). However, it is difficult to estimate the true extent of SARS-CoV-2 infections in Portugal, although a previous study of clinical patients indicated a seropositivity <2.9% (*1*). We report a national, cross-sectional, epidemiologic survey that used quota sampling to quantify more accurately the cumulative number of infected persons in Portugal.

## The Study

We used a convenience quota sampling, quasi-proportional to the population of Portugal in 9 strata: age group (<18, 18‒54, and >55 years of age), each subdivided by population density of place of residence (<60, 60‒500, and >500 persons/km^2^) (Appendix). After a widespread media campaign, we recruited participants by using voluntary registration on a website specifically designed for this study. We obtained informed consent from all participants >16 years of age and from legal guardians for participants <18 years of age. The study was approved by the Ethics Committee of the Centro Académico de Medicina de Lisboa (#350/20, July 30, 2020).

Blood collections and serologic tests were performed by Centro de Medicina Laboratorial Germano de Sousa (Lisbon, Portugal) by using standard procedures. We determined total antibodies against SARS-CoV-2 by using a chemiluminescent immunoassay test (COV2T; Advia Centaur Siemens, https://www.siemens-healthineers.com), which targets the spike protein. This antibody test has a sensitivity of 98.1% and a specificity of 99.9% (*2*), which we used to correct the seroprevalence estimates by using the Rogan‒Gladen estimator (*3*). We used sample weights and poststratified by sex to adjust the seroprevalence, extrapolating from the strata to the whole population (Appendix Tables 1‒4). Participants completed a questionnaire with demographic, clinical, and epidemiologic questions regarding SARS-CoV-2 exposure (Appendix). We use standard statistical analyses to compare results at an α = 0.05 significance.

We enrolled 13,398 participants (55.3% women, age range 1‒92 years) (Appendix Figure 2). Our sample reflected approximately the characteristics of the population in Portugal, except for overrepresentation of women, persons who had higher levels of education, persons living in households that had >1 person, and workers in the education and health sectors (Appendix Tables 5‒7).

We obtained blood samples during September 8‒October 14, 2020; a total of ≈90% were obtained by September 19. Overall seroprevalence was 2.2% (95% CI 2.0%–2.5%; n = 296 positive participants) (Table 1). The differences seen among age groups did not reach statistical significance. We found a higher seroprevalence in regions of high population density (2.9%, 95% CI 2.5%‒3.4%) versus regions of medium population density (1.6%, 95% CI 1.4%‒2.1%) and low population density (1.4%, 95% CI 1.1%‒2.2%) (Appendix Figure 3).

**Table 1:** Prevalence of antibodies against severe acute respiratory syndrome coronavirus 2, by person age, adjusted for sensitivity and specificity, Portugal, September 8‒October 14, 2020

Population density	Seroprevalence, % (95% CI), by age, y			
	<18, n = 2,108	18–54, n = 6,495	>55, n = 4,795	Overall, n = 13,398
Low, n = 2,298	0.6 (0.2‒2.8)	1.5 (0.9‒2.6)	1.7 (1.0‒2.9)	1.4 (1.1‒2.2)
Medium, n = 5,006	1.4 (0.8‒2.7)	1.7 (1.3‒2.4)	1.7 (1.2‒2.5)	1.6 (1.4‒2.1)
High, n = 6,094	3.5 (2.5‒5.0)	3.1 (2.6‒3.9)	2.2 (1.7‒3.1)	2.9 (2.5‒3.4)
Overall	2.4 (1.9‒3.3)	2.3 (2.0‒2.8)	1.9 (1.6‒2.4)	2.2 (2.0‒2.5)

**Figure 1:**
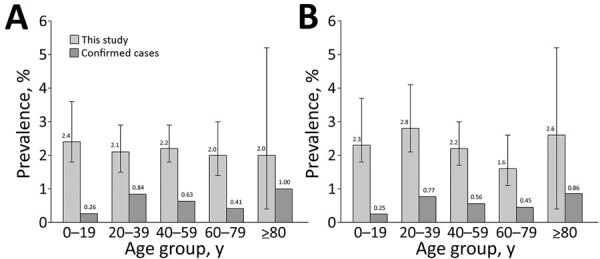
Seroprevalence of antibodies against severe acute respiratory syndrome coronavirus 2, Portugal, compared with official reported confirmed cases, by sex and age. A) Female; B) male. Adjusted seroprevalence measured in this study (numbers above light gray bars) is compared with confirmed cases (numbers above dark gray bars) as a fraction of the corresponding population group (on September 1, 2020). Error bars indicate 95% CIs for estimates. This figure includes different age ranges for consistency with the official data on number of cases by age.

**Figure 2:**
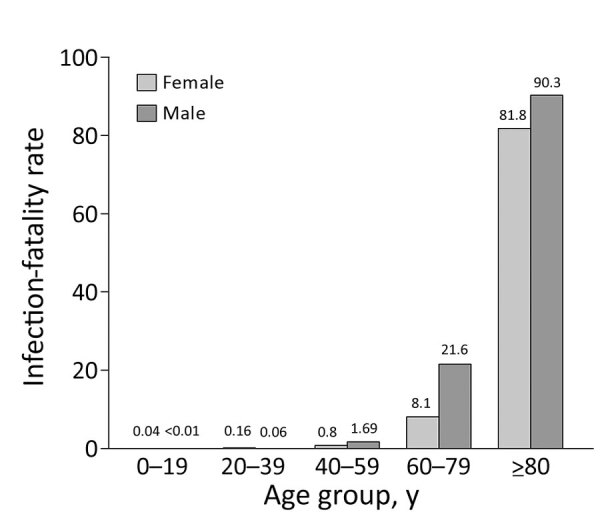
Inferred infection-fatality rate from seroprevalence estimates for antibodies against severe acute respiratory syndrome coronavirus 2, Portugal. We used the registered number of deaths (on September 21, 2020) by age and sex and our prevalence estimates based on seropositivity to infer the infection-fatality rate (Appendix) for more details. Numbers above bars indicate deaths per 1,000 persons.

Comparing the seroprevalence (2.2% corresponds to ≈226,000 persons in Portugal) with the number of official cumulative confirmed cases (55,720 on August 24 and 76,396 on October 1) (*4*), we found a 3–4-fold larger number of persons who had antibodies than those reported infected. This factor varied across age groups; we found an ≈9-fold difference for young participants versus a 2–5-fold difference (depending on sex and age) in middle-age and older participants (Figure 1). With our estimate of cumulative cases, we calculated that the infection-fatality rate varied from <0.2% in younger persons to up to 9.0% in men >80 years of age (Figure 2). The estimated proportion of asymptomatic persons among seropositive persons was 17.4% (95% CI 14.1%‒22.9%); this proportion was much higher for persons <18 years of age (Appendix Table 8).

We found no difference between seropositivity levels in men and women (2.3% vs. 2.1%) (Table 2; Appendix Table 9). There were small differences in seroprevalence by occupation and professional sector; and teleworkers had a lower seroprevalence (1.4%) than nonteleworkers (2.4%) (Table 2). We also did not find differences in seroprevalence for persons who had chronic conditions versus persons who did not (Appendix Table 10). One of the largest differences was between nonsmokers and smokers (2.4% vs. 1.0%) (Table 2).

**Table 2:** Prevalence of antibodies against severe acute respiratory syndrome coronavirus 2, weighted and adjusted for the strata, by sociodemographic, health, and epidemiologic characteristics, Portugal, September 8‒October 14, 2020*

Characteristic	No. participants	Seroprevalence, % (95% CI)
Overall	13,398	2.2 (2.0–2.5)
Sex		
M	5,985	2.3 (2.0–2.8)
F	7,413	2.1 (1.9–2.6)
Persons per household		
1	1,141	2.1 (1.6–4.6)
2‒4	11,139	2.1 (1.9–2.5)
>5	1,069	2.3 (1.9–4.5)
Education		
Less than high school	4,145	2.1 (1.7–2.8)
High school, post-high school, no undergraduate degree	3,373	2.0 (1.8–3.1)
Undergraduate or graduate degree	5,603	2.1 (1.8–2.7)
Other	270	2.9 (2.1–10.6)
Occupation		
Employed	7,584	2.3 (2.0–2.8)
Unemployed	668	2.5 (1.9–5.1)
Student	2,488	2.3 (1.9–3.5)
Retired	1,943	1.6 (1.2–3.0)
Disability	143	0.3 (0.2–3.0)
House worker	223	1.0 (0.5–6.6)
Other	333	3.8 (2.9–9.1)
Professional sector		
Commerce/industry/building	1,324	2.7 (2.2–4.3)
Administration/services	1,930	2.5 (2.0–3.7)
Education	1,351	1.5 (1.1–3.1)
Health	875	3.2 (2.4–5.9)
Transportation	207	3.2 (2.3–10.3)
Other	1,772	1.8 (1.4–3.0)
For employed workers, current working arrangements		
Teleworking		
No	6,480	2.4 (2.2–3.0)
Yes	1,104	1.4 (1.0–3.1)
Physically at work, contact with colleagues		
No	263	2.8 (2.0–8.4)
Yes	6,579	2.3 (2.1–2.9)
Physically at work, contact with the public		
No	4,184	2.3 (1.9–3.0)
Yes	3,400	2.3 (2.0–3.2)
Body mass index, kg/m^2^†		
Underweight and normal weight, <24.99	5,352	1.8 (1.5–2.3)
Overweight, 25.00–29.99	4,166	2.4 (2.0–3.1)
Obese, >30.00	1,766	2.5 (2.0–3.7)
Smoking status		
Non-smoker	9,235	2.4 (2.1–2.9)
Ex-smoker	2,298	2.2 (1.8–3.4)
Smoker	1,862	1.0 (0.9–2.2)
<20 cigarettes/day	1,689	1.1 (0.9–2.4)
>20 cigarettes/day	173	0.0 (0.0–5.2)
Physical exercise		
No	7,590	2.2 (2.0–2.7)
Yes	5,808	2.0 (1.7–2.5)
BCG vaccine		
No	688	2.6 (2.0–5.2)
Yes	10,672	2.2 (2.0–2.6)
Do not know	2,038	2.0 (1.6–3.2)
Chronic disease		
No	9,681	2.2 (2.0–2.6)
Yes	3,717	2.0 (1.6–2.8)
Were you in contact with someone infected?		
No	3,621	0.7 (0.6–1.3)
Yes	1,025	16.2 (14.2–19.3)
Do not know	8,752	1.2 (1.1–1.6)
Where was this potential contact?		
Household	295	23.6 (19.8–30.3)
Work	432	9.6 (7.7–17.2)
Family outside household	210	12.9 (10.2–20.9)
Healthcare institution	48	7.5 (5.2–20.9)
Do not know	40	18.4 (13.8–35.7)
Was someone in your household given a diagnosis of COVID-19?		
No	12,997	1.3 (1.2–1.6)
Yes	401	28.3 (24.5–33.7)
Were you given a diagnosis of suspected COVID-19?		
No	12,729	1.1 (1.0–1.4)
Yes	669	22.7 (19.9–26.8)
If you took a SARS-CoV-2 test, what was the result?		
Positive	136	80.1 (70.4–82.3)
Negative	2,030	2.5 (2.1–4.0)
Inconclusive	27	22.4 (15.4–37.1)
If you took an antibody test before, what was the result?		
Positive	31	62.1 (45.7–69.2)
Negative	219	1.9 (1.4–11.1)
Inconclusive	9	8.0 (4.6–18.8)

*We include the number of participants in the study for reference purposes; these numbers are not adjusted for the population. BCG, Bacillus Calmette–Guérin; COVID-19, coronavirus disease; SARS-CoV-2, severe acute respiratory syndrome coronavirus 2. †Calculated only for adults (>18 years of age).

Of the seropositive participants, 50.0% had never been given a diagnosis as being a case or a suspected case of infection (Appendix Table 11). However, 5% (n = 669) of participants were considered as having a suspected case of COVID-19 before the study (Table 2). This number is consistent with the number of suspected cases, which the national health authorities reported until August 16, 2020, two weeks before the start of our study, when there were a cumulative 468,937 suspected cases (only 54,102 confirmed), corresponding to 4.6% of the population of Portugal.

## Conclusions

We found a seroprevalence of 2.2% for antibodies against SARS-CoV-2 in the population of Portugal, which was lower than that in a previous smaller study (*1*). Our results suggest that 3–4-fold as many persons were infected by SARS-CoV-2 than was officially reported by health authorities. This factor is consistent with, albeit somewhat smaller than, results reported in other national seroprevalence studies (*5**–**7*) and varied across age groups.

The higher seroprevalence in younger participants is in contrast to the official number of confirmed cases in Portugal, where there is a higher prevalence in older persons (*4*), possibly because younger persons tend to have milder disease, often asymptomatic (*8**,**9*). We found that ≈40% of infections were asymptomatic in persons <18 years of age, whereas this proportion was much lower in older persons. Overall, if only ≈20% of cases are asymptomatic, a question is why so many cases go undetected even with higher testing rates, as in Portugal before our study (*10*). This discrepancy highlights the public health relevance of conducting seroprevalence studies.

Despite a similar prevalence, we found that the infection-fatality rate for men was higher than that for women, particularly in persons >40 years of age. The rate was more than twice as large for persons 60–79 years of age (2.16%) than for the overall group (0.81%). These values are consistent with those reported in Spain (*11*) and include only confirmed COVID-19 deaths, not all excess deaths during this period (*12*).

A limitation of our study is that we used quota sampling, relying on volunteers for the study. We chose our method of recruitment to achieve a fast enrollment process because, during a pandemic, the number of persons positive for antibodies is changing continuously. We reasoned that such changes could bias the study more than the method of recruitment. In addition, even studies with a fully random sample often have a large fraction of persons refusing to participate or unable to be contacted (*6**,**13*). Another limitation is that we used relatively large intervals for age groups. A more fine-grained stratification, along with other variables (e.g., sex), would be more representative of epidemiologic and clinical aspects of SARS-CoV-2, but would require a larger sample. We also did not correct for potential seroreversion (*14*), which reduces the fraction of seropositive results in relation to the actual number of infections and lowers the estimated infection-fatality rate. However, we expect seroreversion over the short 6-month period covered by our study to be minimal (*15*). These potential limitations are common to most seroprevalence studies but do not limit the need for conducting these studies during the evolving pandemic.

Overall, our results demonstrate a low incidence of SARS-CoV-2 during the first wave (spring and summer 2020) of the pandemic in Portugal. This incidence probably resulted from control measures that were relatively successful, in comparison with other countries with higher seroprevalence over similar (or shorter) periods (*6*).

Appendix Additional information on prevalence of SARS-CoV-2 antibody after first 6 months of COVID-19 pandemic, Portugal.
