# Supplementary material for: Prevalence of SARS-CoV-2 Antibodies after First 6 Months of COVID-19 Pandemic, Portugal
Source: Emerg Infect Dis. 2021 Nov;27(11):2878–81. doi: 10.3201/eid2711.210636 (PMC8544986; doi:10.3201/eid2711.210636)
Supplement: Appendix — Additional information on prevalence of SARS-CoV-2 antibody after first 6 months of COVID-19 pandemic, Portugal. [file 21-0636-Techapp-s1.pdf]

Article DOI: https://doi.org/10.3201/eid2711.210636

Prevalence of SARS-CoV-2 Antibody after First 6 Months of the COVID-19 Pandemic, Portugal

Appendix

Study Context

It is challenging to determine the true extent of SARS-CoV-2 infection in different countries due to different testing programs and capacities, and variations in the fraction of asymptomatic infections (*1–4*). In this context, seroepidemiologic surveys are a powerful tool to help estimate the true prevalence of infection in a given population. There have been multiple seroprevalence studies for SARS-CoV-2 in different settings around the world (*5–11*), summarized in several meta-analyses-type studies (*12–14*), but there have been few national-level studies (*3,15–20*).

Portugal, a country of ~10.3 million inhabitants, was moderately affected by SARS-CoV-2 infection during the first wave of the pandemic in March through September, 2020. The burden of disease, total number of recorded cases and deaths, was similar or smaller than other European countries of similar size at this time of the pandemic (Appendix Figure 1). In addition, hospital capacity was never reached, and the National health system was able to respond to the crisis without the same level of issues seen in other regions (e.g., Spain, Italy, and New York). In particular, the government reacted swiftly to impose public health measures to try to curb the spread of infection starting on March 16, 2020, when there were 448 officially registered cases and one death attributed to COVID-19 (*21*). These measures included the closure of all levels of schools (pre-K to university) on March 16 until June 1, (when lower levels of schools re-opened), the imposition of a national emergency state and a lockdown from March 18 to May 4. This lockdown entailed special permissions to be outside the home, with few exceptions, no travel between counties, compulsory teleworking when feasible, all non-essential commerce and services closed, restaurants only with take-away. These measures led to a peak and decrease in the number of daily cases starting on April 12. Over the Summer holiday period (July and

August), the number of cases was somewhat elevated, but steady, with a daily average of 255 cases.

As of June 2, 2020, Portugal was one of the ten countries in the world with highest levels of testing in per capita terms (*22*). This notwithstanding, the potential for asymptomatic infections makes it difficult to estimate the true extent of SARS-CoV-2 infections in Portugal after the first phase of the pandemic, although an earlier, more limited, study estimated seroprevalence at 2.9% (*23*).

Calculation of Sample Size

The sample was stratified by age groups (<18, 18 to 54, ≥55 years old) crossed by population density of the place of residence (<60; 60 to 500; >500 persons/km^2). These strata were chosen for epidemiologic reasons. Age is a major factor in COVID-19 severity, and the three age groups were chosen based on cut-offs proposed in a vaccine trial (*24*). Population density is a major factor in the transmission of infectious diseases, and the three groups were chosen to have a good balance between number of counties sampled and total population in each density strata. At the same time, we strived to keep the total number of strata at <10, to reduce the logistical complexities and sample size associated with more strata. The overall sample size was determined by assuming low prevalence in each of the nine strata, between 0.1% and 3%, with lower levels in the regions of low population density. We also defined a relative error margin of 15% for the global prevalence estimate (i.e., error margin could be at most 15% of the observed prevalence). In addition, we assumed that the test to be used would have 99% sensitivity and 98.7% specificity. Using the test characteristics changes the expected fraction of positive actually observed in the study (see below). We then used these corrected seroprevalence values and Cochrane's formula for proportional allocation to estimate sample size in stratified populations (*25*), and obtained a sample size of at least 11,241 persons divided proportionally among the 9 strata mentioned. To guarantee precision in the lowest population density regions, where prevalence was expected to be lower, the sample size in those strata (each of the 3 age groups) was increased by 50% of the value calculated. Thus, the final sample is no longer proportional to the population. The total sample size should be at least 11,994 persons distributed according to Appendix Table 1. To achieve the required allocation by population density, the 308 counties of Portugal (including both the Madeira and Azores archipelagos) were subdivided into the three levels of population density and 104 were randomly selected to be sampled, among all

counties with a collection laboratory, and with the number of persons in each age group per county as prescribed in (Appendix Table 2).

Recruitment of Study Participants

For logistical reasons, we recruited volunteers to this study, according with the quotas defined (Appendix Table 2). Thus, this study uses a convenience sample. To achieve the needed number of participants from all of Portugal, we developed a communication and study dissemination strategy with several layers. One month before the beginning of the study, the main media groups in Portugal were contacted to aid in the broadcasting of this project. Media Capital, a large group representing 2 TV channels (TVI and TVI 24, over the air broadcast and cable, respectively) and several radio stations (Rádio Comercial, M80, Cidade FM) with National coverage, promptly joined in, promoting short campaign videos featuring TV and News hosts in teasers aired at the beginning of the recruitment. Additionally, a press release containing all the information about the study and how to participate, was widely distributed to the Portuguese media, 1 week before the beginning of the study (with embargo). This enabled several news pieces to be prepared in advance and released on the first day of the study. During September 8–30, a total of 296 news clippings, reaching all regions of Portugal, about the study were registered.

We also implemented a campaign of leaflet distribution and poster advertisements, through one of the funding partners of this study: Jeronimo Martins Group, which owns one of the largest supermarket chains in Portugal (Pingo Doce), again with implantation in all regions of Portugal. To help disseminate the study to a larger audience, a leaflet was prepared and distributed in the Pingo Doce stores across the country. At the entrance of the stores, advertisement posters were visible to all the clients. Additionally, advertisement posters were distributed to the 314 participating Germano de Sousa laboratories.

Finally, we used social media, including a short video (https://www.youtube.com/watch?v = TiKMz-Ne9bo) and specifically designed materials were produced for the communication of the project through the institutional social media channels (Facebook, Instagram, LinkedIn, Twitter, and YouTube), again reaching a wide audience. We also had an email and phone lines dedicated to the study, through which interested persons could reach us for help in registration or information about the study.

All participants were recruited by voluntary registration through a Web site specifically designed for the study. To help citizens with fewer digital skills, the enrollment could be done directly at one of the 314 participating blood collection laboratories (Germano de Sousa Laboratories), where the local technicians could support and assist in the process of registration through the Web site. Participants were not given any compensation beyond being informed of their serologic status. Participants were excluded only if they had any contraindication for phlebotomy. Prior diagnosis of SARS-CoV-2 infection was not an exclusion criterion.

Blood Collection and Serologic Tests

All blood collections and serologic tests were done by Centro de Medicina Laboratorial Germano de Sousa (CMLGS), an ISO 9001:2015 certified private laboratory, which performs serologic tests for SARS-CoV-2 according to the clinical guidelines issued by the Directorate-General of Health (DGS), within the Portuguese Ministry of Health. CMLGS has a national network of collection sites, of which 314 were involved in this study. This network enabled blood collection from the participants, wherever it was most convenient for them, typically in their area of residence. Each participant donated 7–9 mL of blood collected into tubes with separation gel and without any anti-coagulant, for a 4–5 mL of serum sample, obtained by centrifugation. All samples were transported to the central laboratory, according to usual procedures, where they were assayed.

Blood samples were assayed for total antibodies against SARS-CoV-2 by using the Siemens SARS-CoV-2 Total (COV2T) (Advia Centaur Siemens, Siemens Healthcare, Portugal), a chemiluminescent immunoassay test targeting the spike protein. Positive samples were stored at Biobanco-iMM, Lisbon Academic Medical Center.

Epidemiologic Questionnaire and Outcomes

All participants completed a questionnaire with sociodemographic, general health and clinical/epidemiologic questions regarding SARS-CoV-2 exposure, including symptoms of interest. The full (translated) questionnaire is presented near the end of this Appendix. The questionnaire was in Portuguese (the overwhelmingly dominant language in Portugal), and it was tested beforehand in a study of the University of Lisbon, involving ≈2,500 persons (mostly staff). The questionnaire was completed at enrollment, and it was the only way participants could get a code to perform the free blood draw, within 2 weeks.

The primary outcome was the proportion of serologic positive cases defined as the fraction of participants who were positive for SARS-CoV-2 specific total antibodies: overall and stratified by age and population density. The secondary outcomes included the proportion of serologic positive cases without any symptoms of interest (asymptomatic cases); or with <3 symptoms and without sudden loss of smell or taste (pauci-symptomatic cases as defined) (*3*). The symptoms of interest reported by participants in the questionnaire were: loss of smell/taste, fever, chills, cough (dry or with mucus), muscle or joint pain, sore throat, headaches, general weakness/tiredness, respiratory difficulty, gastrointestinal issues (vomit, nausea, diarrhea), loss of appetite, rashes, rhinorrhea, or loss of consciousness.

Finally, the associations between antibody positivity and the sociodemographic, health and epidemiologic characteristics of the participants were explored. We included questions about education, household size, occupation, chronic disease conditions, body mass index, exercise, smoking habits, influenza and Bacille Calmette-Guerin (BCG) vaccine (against tuberculosis), contact with persons who had COVID-19, previous tests for SARS-CoV-2, among others (see questionnaire).

Adjustment of Seroprevalence for Sample Weights

To extrapolate our results for the entire population, sample seroprevalences were adjusted based on official estimates for the resident population, per quinquennial age group, in each county of Portugal as of December 31, 2019 (*26*), and further adjusted for the overrepresentation of women by post-stratifying the sample on sex. The weights for each of the 9 study strata divided by sex are presented (Appendix Table 3).

Due to the low values of seroprevalences, specific methods were favored in the calculation of upper and lower limits of the CIs, in detriment of methods based on the normal approximation to the binomial distribution. In particular, Jeffreys CIs for a proportion were used at the strata level (*27*). To calculate CIs for aggregated strata (i.e., marginal values), we used the exact limiting terms for the binomial parameter adapted for weighted proportions (*28*).

Correcting Seroprevalence Estimates with Test Sensitivity and Specificity

The total antibody test has a sensitivity, from 14 days post-infection, of 98.1% (based on 536 positive samples); and a specificity of 99.9% (based on 994 samples) (*29*).

The seroprevalence observed in our weighted sample was adjusted taking into consideration the sensitivity and specificity of the tests by using the Rogan–Gladen estimator (*30,31*)

$$P_{adj} = \frac{P_m + S_p - 1}{S + S_p - 1},$$

where P_m is the measured prevalence and P_{adj} is the final adjusted prevalence, as reported in the main text, with the test specificity Sp and sensitivity S.

Correcting the Asymptomatic and Pauci-Symptomatic Prevalence Estimates with Test Sensitivity and Specificity

The proportion of asymptomatic observed in our weighted sample was adjusted taking into consideration the sensitivity and specificity of the tests, by using the following formula, deduced by applying standard results from probability theory (see the section at the end of this Appendix),

$$A_{adj} = \frac{AP_m - (1 - S_p)A_S}{P_m + S_p - 1},$$

where A is the observed weighted proportion of asymptomatic in the seropositive participants P_m is the measured seroprevalence, A_S is the observed proportion of asymptomatic in the full sample, Sp is the test specificity and A_{adj} is the final adjusted proportion of asymptomatic, as reported in the main text. Similarly, the proportion of pauci-symptomatic observed in our weighted sample was adjusted taking into consideration the sensitivity and specificity of the tests.

Comparison to Official Reported Cases

To compare our seroprevalence results with official reported cases, we used cutoffs in 10-year age groups, which is how the official statistics are presented. For each of the age intervals (Figure 1 of the main text), we calculated the seroprevalence in Portugal by sex and compared it to the fraction of reported cases, as a proportion of the respective age-sex population in Portugal. We then calculated the multiplier corresponding to how many more cases our seroprevalence study found compared with those officially reported. For this analysis, we used the number of reported cases on September 1, 2020 (*21*). We use this date to account for some time between

infection and seroconversion, which has been reported to take ≈2 weeks (*32–34*). Since 90% of blood samples from participants were collected between September 8 and September 19, 2020, the chosen date is good for this comparison. Note that incidence was stable: ≈50 cases/million persons/day in early September (Appendix Figure 1).

Calculation of Infection-Fatality Rates

We used the official number of deaths due to COVID-19 by age and sex divided by our estimated number of cases in the total population to obtain the infection-fatality rate (IFR). Again, we used cutoffs in 10-year age groups, which is how the official statistics are presented. In addition, we took into account the typical delay between infection and death, which we assumed to be ≈3 weeks (*35,36*). If we assume that we are estimating infections up to September 1, 2020 (see above), then we should calculate IFR with death data from September 21. We note that there are more sophisticated ways to take into account the distribution of times until death (*37,38*), but here for simplicity and for lack of data on that distribution, we just calculate the quotient of deaths on September 21 by the total number of estimated infected in our study. Thus, this is only an approximation to the IFR, albeit likely a good one, because the numbers of cases and deaths were relatively low around these dates.

Statistical Analyses

We used the χ^2 test to compare categorical variables (e.g., distribution of the number of positive and negative participants with a given symptom), except when the numbers in some groups were low, when we used the Fisher exact test. We used logistic regression to analyze the effect of smoking status on prevalence of seropositivity, controlling for sex and age. For this, we used the *survey* package of R (*39*). We did not input any missing values.

All statistical analyses were two-sided, the significance level was $\alpha = 0.05$, and reported CIs are at the 95% level. Statistical analyses were done by using SAS version 9.4 (SAS Institute Inc, Cary, NC, USA) and R version 3.6.1 (R Foundation for Statistical Computing, Vienna, Austria).

Sample Representativeness

Overall, comparing with the sociodemographic characteristics of the Portuguese population, we found an overrepresentation in the education and health sectors (36% of employees in the sample, compared with 19% in the population). This had an impact on some

characteristics of the 18–54 age group: more women, more graduates and fewer persons living alone than in the global population of these ages. We present the characterization of this sample regarding sociodemographic and health characteristic (Appendix Tables 5–7).

SARS-CoV-2 Antibody Seroprevalence in the Population in Portugal

As we mentioned in the main text, the differences in seroprevalence across age groups were not statistically significant. However, this difference was highly dependent on population density, with the lowest observed seroprevalence in the youngest group in low population density areas (0.6%) and the highest seroprevalence also in the youngest group, but in high population density areas, with a point estimate 6 times higher (3.5%) (Table 1, main text).

After adjusting for sensitivity and specificity, the estimated proportion of asymptomatic among seropositive was 17.4% (95% CI 14.1%–22.9%), and the prevalence of asymptomatic cases was much higher in persons <18 years of age (Appendix Table 8). If we consider pauci-symptomatic cases, which also includes asymptomatic cases, the proportion among seropositive persons increases to 19.9% (95% CI 16.1%–25.4%), also with significantly higher values for persons <18 years old (39.6%) (Appendix Table 8).

Demographic, Health, and Epidemiologic Determinants of Seroprevalence

We found no difference between seropositivity levels in men and women (2.3% vs. 2.1%) (Table 2; Appendix Table 9). In terms of occupation, there were small differences in seroprevalence between employed persons (2.3%), unemployed persons (2.5%), or students (2.3%). However, for retired persons, we found a lower seroprevalence level (1.6%). It is noteworthy that healthcare professionals (3.2%) and transport sector workers (3.2%) had higher levels of seroprevalence than other workers, such as persons in commerce, industry, education, services, or construction. About 15% (n = 1,104) of employed participants reported that they were teleworking, and teleworkers show a lower seroprevalence (1.4%) than non-teleworkers (2.4%), independently of whether the latter had contact with other persons at work (Table 2; Appendix Table 9).

We also enquired about health conditions and 27.7% (n = 3,717) participants reported at least 1 chronic condition, but we found no differences in seroprevalence for persons with or without such conditions (Appendix Table 10). However, there was, a significant difference (p = 0.002) between persons who do not smoke (n = 9,235 participants) and those who smoke (n =

1,862 participants), with an estimated higher seroprevalence among persons who do not smoke (2.4%; 95% CI 2.1%–2.9%) compared with persons who smoked (1.0%; 95% CI 0.9%–2.2%) (Table 2 in the main text). When we considered together ex-smokers and smokers, the seroprevalence in this group of ever smokers was 1.7% (95% CI 1.4%–2.5%). Ex-smokers seem to have a prevalence closer to non-smokers than to that of smokers. Smokers (median age = 47 years) were older than non-smokers (median age = 41 years), and as mentioned above, older participants had a lower prevalence. In addition, smoking status differs by sex; more men smoked than women ($p = 0.001$). Thus, we performed a logistic regression of seroprevalence on smoking status controlling for the possible confounding factors of age and sex. In this analysis, smokers still had a significantly lower seroprevalence ($p = 0.003$). Further analyses of this result indicated that women were the main drivers for this difference in seroprevalence between smokers and non-smokers.

We also considered other health-related variables. For example, there was no difference in seroprevalence among participants who practice regular exercise versus those who do not. We also enquired about Bacille Calmette-Guerin (BCG) status (a vaccine against tuberculosis). In our study, 688 participants reported not taking this vaccine versus 10,672 who did, and seroprevalence was not statistically different between these groups (Appendix Table 10). Finally, although we found a slight over-representation of overweight and obese persons in seropositive when compared with seronegative participants, this result was not statistically significant (Table 2).

Among participants who believed that they had been in contact with an infected person, prevalence was 16.2% (95% CI 14.2%–19.3%), and most of these contacts were reported to be at work. Prevalence among participants, who had someone infected in their household, was 28.3% (95% CI 24.5%–33.7%) (Table 2). Of the 401 participants who indicated that someone in their household had been given a diagnosis of COVID-19, 71.3% (n = 286) were seronegative, and presumably were not infected by their household contact.

Clinical Comparison of Seropositive with Seronegative Cases

Based on the clinical questionnaire, the symptoms with largest differences in reporting between seropositive and seronegative participants were loss of taste (42.4% of seropositive participants vs. only 2.8% of seronegative participants), loss of smell (39.3% vs. 2.0%), general

weakness (38.6% vs. 11.5%), fever (temperature) >38°C (32.9% vs. 6.1%), feeling tired (51.9% vs. 27.6%), muscular or joint pain (49.2% vs. 25.3%), and lack of appetite (28.5% vs. 6.8%), all of which were significantly more common in seropositive participants ($p<0.0001$, for all of these symptoms) (Appendix Table 10). For persons who had loss of smell or loss of taste, we estimated seroprevalences of 31.2% (95% CI 27.1%–37.1%) and 27.7% (95% CI 23.7%–32.8%). These are the symptoms, and the subgroups of participants, in whom prevalence is the highest, indicating a good positive predictive value.

A total of 50.0% of seropositive participants had never been given a diagnosis of having a case or suspected case of infection (Appendix Table 11), and 42.9% of them had never taken a diagnostic test for SARS-CoV-2. Of the 169 seropositive persons who took such a test, 29.0% (n = 49) had a negative result. Conversely, when seronegative participants were analyzed, 4.0% (n = 521) were considered to have had a suspicious case at some point before this study. However, most of these suspicions were not confirmed because of those 521 participants, 435 actually had a reverse transcription PCR (RT-PCR) for SARS-CoV-2 and only 24 had a positive result. Altogether, among the 2,025 seronegative participants who had an RT-PCR before our study, 1.2% (n = 24) were positive. These tests were performed a median of 88 days (minimum 12 days and maximum 186 days) before the study.

Results in Context

We found an overall prevalence of 2.2% of persons positive for antibodies against SARS-CoV-2 in the population of Portugal. This prevalence is was lower than that for an earlier smaller study, using samples from persons who were tested in clinical laboratories for non–SARS-CoV-2 reasons, which showed a seroprevalence ≤2.9% (*23*). Our results suggest that there were 3–4-fold as many persons infected by SARS-CoV-2 than those officially reported by health authorities. However, this factor varied across age groups, being ≈9-fold among younger persons (<18 years of age, both males and females). This result is striking because it contradicts the recent suggestion that young persons might have a lower susceptibility to infection compared with adults (*40*). However, other seroprevalence studies also reported this large discrepancy between seropositive young persons and official reported cases (*41*).

We found that ≈40% of infections were asymptomatic in persons <18 years old, whereas this proportion was much lower in older persons. However, we note that, in this study, a

participant was considered asymptomatic if she or he had not experienced any of the listed symptoms since the beginning of the pandemic (i.e., within a period of 6 months). Thus, the percentage of asymptomatic infection is probably an underestimate, although it is consistent with other values reported (*1–4*).

Spain, the only country with which Portugal has land borders, reported 5% seroprevalence in a study done 4 months before ours (*3*). The dire situation observed early on in some regions and hospitals of Spain had a profound influence in the nonpharmaceutical control measures imposed by the Portuguese authorities, and these seemed to have been successful in controlling the spread of infection.

We found similar seroprevalence estimates for men (2.3%) and women (2.1%), which translates into more women having been infected than men because ≈53% of the population in Portugal are women (*42*), and it is also consistent with the number of confirmed cases, in which women had ≈54% of the cases. Our results also show that retired (older) persons, who might take more care not to expose themselves to the virus, had lower seroprevalence (1.6%) than other groups. Among those working, teleworking resulted in lower seroprevalence, when compared with persons physically present at their work locations. In addition, in workers of certain sectors (such as healthcare or transportation) seroprevalence was higher. Some of these differences did not reach statistical significance, but are suggestive of differences in risk for acquiring infections. In this respect, we did not find differences in seroprevalence among persons with and without previous chronic health conditions. Given the widespread knowledge that some chronic conditions are major risk factors for severe disease, one might expect persons who had comorbidities to take extra precautions to avoid infection. However, our data do not support this expectation.

We were also able to analyze 2 controversial issues related to the risk for infection. First there have been some reports of a link between smoking and risk for SARS-CoV-2 infection (or COVID-19 severity). A few studies looked at risk for infection (asymptomatic, mild, or severe), including an ecologic meta-analysis (*43*), and a study of an outbreak on an aircraft carrier (*44*), indicating a potential protective effect of smoking. Conversely, a large cross-sectional study based on a symptom app indicated an increased risk for (symptomatic) infection for smokers (*45*). In our population-based study, with self-reported smoking status, we found a lower

seroprevalence in smokers (1.0%) vs. non-smokers (2.4%), which was one of the most robust differences, even when accounting for sex and age of the participants. Women were the drivers of this finding, and if we analyzed only the men, we found that the difference in prevalence between smokers and non-smokers was no longer significant. Although these results were clear, it is essential to stress that smoking is a well-known risk factor for many other pathologies, most more pathogenic than SARS-CoV-2 infection (*46*). In addition, it is probable that once infected, smokers have a worse prognosis (*47*). Thus, our findings should be interpreted cautiously.

Another debated issue is the suggestion that the BCG vaccine might be protective against infection (*48*), which led to some ongoing clinical trials to analyze that hypothesis (*49*). In our study, there was a slightly increased prevalence of total SARS-CoV-2 antibodies in those reporting not taking the BCG vaccine (2.6%) versus participants who had taken the vaccine before (2.2%), which was not statistically significant, but it is consistent with a recent result (*50*). We note that only a small percentage of persons (≈6%) report not taking this vaccine (excluding those that did not know their BCG status), which is in accordance with the recommendation of universal vaccination in Portugal until 2016.

Some seronegative patients reported that they had been given a diagnosis of having a suspicious case of COVID-19. However, almost none of these cases were actually confirmed by PCR. This finding is probably caused by heightened awareness of the infection, leading to many spurious diagnoses. According to the responses of participants, >60% of these suspicious cases were diagnosed by using SNS24, a National Health Service telephone line managed by the government as a first line of medical advice (not just during the pandemic). The national health authorities reported the number of suspected cases in their daily briefings until August 16, 2020 (*21*). On that day, 2 weeks before the start of our study, there were 468,937 suspected cases, which corresponds to 4.6% of the ≈10.3 million persons in Portugal. The number of suspicious diagnosis in our sample is consistent with that value. However, there were 24 seronegative persons who reported having a positive RT-PCR result before our study.

There are several possible explanations for this observation. These persons could have true negative results (e.g., persons who did not yet have antibodies, persons who might have lost antibodies (seroreversion), or persons who had a false-positive RT PCR result). Alternatively, they could be persons who had false-negative results in our antibody test. In any case, when

correcting our prevalence estimates with the sensitivity and specificity of the test, we are (up to a point) taking into account these potential false-negative results in the antibody test.

As stated in the main text, our study has some limitations. We used quota sampling, relying on volunteers for the study. Thus, our sample might not reflect the population of Portugal in some demographic/epidemiologic respects. We stratified the study and sampled over counties in Portugal to at least have an appropriate representation over these variables (age and population density). In addition, we checked sex distribution by strata and found a distortion in the 18–54 years age group , for all density levels, leading us to post-stratify by sex, despite the resulting larger imprecision in the estimates. However, there is always the possibility that access to the internet, interest in finding serostatus results, and other factors bias the sample of participants. In this regard, it is useful to note that other sample characteristics that deviated from the population statistics, such as education level or household size, were not associated with seroprevalence. One reason we chose our method of enrollment was to achieve a fast enrollment process. During an infection outbreak, the number of persons infected, who eventually will seroconvert, is changing continuously. This process is different from other study situations in which the outcome is more stable (e.g., chronic conditions, behavior, or opinions). If the study (i.e., enrollment) takes too long, then large changes in prevalence during the study period are possible, and it is unclear how to associate the prevalence estimate with a given time period. We reasoned that the occurrence of such changes could bias the study more than the method of recruitment. In addition, we note that studies designed to have a fully random sample often end up with a large fraction of persons not participating (e.g., refusing to participate or could not be contacted), negating the objective of that design choice (*3,16*). Another limitation is that we used relatively large intervals for age groups. Likely, a more fine-grained stratification (e.g., 0–5, 6–10, 11–20, 21–50, 51–60, 61–70, 71–80, >80 years) would be more representative of epidemiologic and clinical aspects of SARS-CoV-2. However, such stratification, as well as adding other variables (e.g., biologic sex), would need a much larger sample size.

Our study was also based on a self-reporting questionnaire, often retrospectively, especially for such issues as past symptoms and behaviors, and we cannot exclude errors in this reporting. We did recontact persons who consented and for whom there were inconsistencies in the questionnaire results that were clear obvious mistakes. In addition, in a study of seroprevalence, there are always potential issues of assay imprecision, which we attempted to

correct on the basis of published sensitivity and specificity. Finally, we did not correct for potential seroreversion, which has been suggested (*51–53*). This phenomenon would reduce the fraction of seropositive persons detected in our study in relation to the actual number of past infections, which would also lower the estimated IFR. We note that this study was conducted 6 months after the start of the pandemic in Portugal, and persons were infected at various times within that period. Several studies, including our own, have now demonstrated that antibodies to SARS-CoV-2 are often detectable for >6 months (*6,54–58*). Overall, we expect seroreversion to have a small impact on our results. However, it is not known if age, severity of disease, or other characteristics of the infected person affect how long antibodies will be detectable after infection. We emphasize that some or all of these potential limitations are common to essentially all seroprevalence studies, and do not limit the usefulness of our study during the evolving pandemic.

Despite these issues, our study demonstrated a low prevalence of SARS-CoV-2 exposure in the Portuguese population during the first wave of the pandemic, between March and September 2020. This study sets the groundwork for continued longitudinal monitoring of the evolution of seroprevalence levels in Portugal.

References

1. Beale S, Hayward A, Shallcross L, Aldridge RW, Fragaszy E. A rapid review and meta-analysis of the asymptomatic proportion of PCR-confirmed SARS-CoV-2 infections in community settings (version 1; peer review: 1 approved with reservations). Wellcome Open Res. 2020;5:266 [cited 2021 Aug 16]. https://doi.org/10.12688/wellcomeopenres.16387.1

2. Byambasuren O, Cardona M, Bell K, Clark J, McLaws M-L, Glasziou P, et al. P. Estimating the extent of asymptomatic COVID-19 and its potential for community transmission: systematic review and meta-analysis. Journal of the Association of Medical Microbiology and Infectious Disease Canada. 2020;5:223–34. https://doi.org/10.3138/jammi-2020-0030

3. Pollán M, Pérez-Gómez B, Pastor-Barriuso R, Oteo J, Hernán MA, Pérez-Olmeda M, et al.; ENE-COVID Study Group. Prevalence of SARS-CoV-2 in Spain (ENE-COVID): a nationwide, population-based seroepidemiological study. Lancet. 2020;396:535–44. PubMed https://doi.org/10.1016/S0140-6736(20)31483-5

4. Syangtan G, Bista S, Dawadi P, Rayamajhee B, Shrestha LB, Tuladhar R, et al. Asymptomatic SARS-CoV-2 carriers: a systematic review and meta-analysis. Front Public Health. 2021;8:587374. PubMed https://doi.org/10.3389/fpubh.2020.587374

5. Capai L, Ayhan N, Masse S, Canarelli J, Priet S, Simeoni MH, et al. Seroprevalence of SARS-CoV-2 IgG antibodies in Corsica (France), April and June 2020. J Clin Med. 2020;9:E3569. PubMed https://doi.org/10.3390/jcm9113569

6. Figueiredo-Campos P, Blankenhaus B, Mota C, Gomes A, Serrano M, Ariotti S, et al. Seroprevalence of anti-SARS-CoV-2 antibodies in COVID-19 patients and healthy volunteers up to 6 months post disease onset. Eur J Immunol. 2020;50:2025–40. PubMed https://doi.org/10.1002/eji.202048970

7. Fischer B, Knabbe C, Vollmer T. SARS-CoV-2 IgG seroprevalence in blood donors located in three different federal states, Germany, March to June 2020. Euro Surveill. 2020;25:2001285. PubMed https://doi.org/10.2807/1560-7917.ES.2020.25.28.2001285

8. Jespersen S, Mikkelsen S, Greve T, Kaspersen KA, Tolstrup M, Boldsen JK, et al. SARS-CoV-2 seroprevalence survey among 17,971 healthcare and administrative personnel at hospitals, pre-hospital services, and specialist practitioners in the Central Denmark Region. Clin Infect Dis. 2020;ciaa1471. PubMed https://doi.org/10.1093/cid/ciaa1471

9. Rosenberg ES, Tesoriero JM, Rosenthal EM, Chung R, Barranco MA, Styer LM, et al. Cumulative incidence and diagnosis of SARS-CoV-2 infection in New York. Ann Epidemiol. 2020;48:23–29.e4. PubMed https://doi.org/10.1016/j.annepidem.2020.06.004

10. Stringhini S, Wisniak A, Piumatti G, Azman AS, Lauer SA, Baysson H, et al. Seroprevalence of anti-SARS-CoV-2 IgG antibodies in Geneva, Switzerland (SEROCoV-POP): a population-based study. Lancet. 2020;396:313–9. PubMed https://doi.org/10.1016/S0140-6736(20)31304-0

11. Waterfield T, Watson C, Moore R, Ferris K, Tonry C, Watt A, et al. Seroprevalence of SARS-CoV-2 antibodies in children: a prospective multicentre cohort study. Arch Dis Child. 2020. PubMed

12. Arora RK, Joseph A, Van Wyk J, Rocco S, Atmaja A, May E, et al. SeroTracker: a global SARS-CoV-2 seroprevalence dashboard. Lancet Infect Dis. 2020. PubMed

13. Lai CC, Wang JH, Hsueh PR. Population-based seroprevalence surveys of anti-SARS-CoV-2 antibody: an up-to-date review. Int J Infect Dis. 2020;101:314–22. PubMed https://doi.org/10.1016/j.ijid.2020.10.011

14. Rostami A. Sepidarkish M. Leeflang MM, Riahi SM. Nourollahpour Shiadeh M, Esfandyari S, et al. SARS-CoV-2 seroprevalence worldwide: a systematic review and meta-analysis. Clin Microbiol Infect. 2020. PubMed https://doi.org/10.1016/j.cmi.2020.10.020

15. Gudbjartsson DF, Norddahl GL, Melsted P, Gunnarsdottir K, Holm H, Eythorsson E, et al. Humoral immune response to SARS-CoV-2 in Iceland. N Engl J Med. 2020;383:1724–34. PubMed https://doi.org/10.1056/NEJMoa2026116

16. Hallal PC, Hartwig FP, Horta BL, Silveira MF, Struchiner CJ, Vidaletti LP, et al. SARS-CoV-2 antibody prevalence in Brazil: results from two successive nationwide serological household surveys. Lancet Glob Health. 2020;8:e1390–8. PubMed https://doi.org/10.1016/S2214-109X(20)30387-9

17. Poustchi H, Darvishian M, Mohammadi Z, Shayanrad A, Delavari A, Bahadorimonfared A, et al. SARS-CoV-2 antibody seroprevalence in the general population and high-risk occupational groups across 18 cities in Iran: a population-based cross-sectional study. Lancet Infect Dis. 2020. PubMed

18. Vos ERA, den Hartog G, Schepp RM, Kaaijk P, van Vliet J, Helm K, et al. Nationwide seroprevalence of SARS-CoV-2 and identification of risk factors in the general population of the Netherlands during the first epidemic wave. J Epidemiol Community Health. 2020;jech-2020-215678. PubMed

19. Research Luxembourg. CON-VINCE [cited 2021 Aug 12]. https://researchluxembourg.lu/covid-19-taskforce/con-vince

20. Petersen MS, Strøm M, Christiansen DH, Fjallsbak JP, Eliasen EH, Johansen M, et al. Seroprevalence of SARS-CoV-2-Specific Antibodies, Faroe Islands. Emerg Infect Dis. 2020;26:2761–3. PubMed https://doi.org/10.3201/eid2611.202736

21. Portuguese Ministry of Health. Status report – COVID-19 [in Portuguese] [cited 2021 Jan 14]. https://covid19.min-saude.pt/relatorio-de-situacao/

22. Triunfol M. High COVID-19 testing rate in Portugal. Lancet Infect Dis. 2020;20:783. PubMed https://doi.org/10.1016/S1473-3099(20)30499-0

23. Kislaya I, Gonçalves P, Barreto M, Sousa R, Garcia AC, Matos R, et al.; ISNCOVID-19 Group. Seroprevalence of SARS-CoV-2 infection in Portugal in May–July 2020: results of the first national serological survey (ISNCOVID-19). Acta Med Port. 2021;34:87–94. PubMed https://doi.org/10.20344/amp.15122

24. US National Library of Medicine. Study to describe the safety Tolerability, immunogenicity, and efficacy of RNA vaccine candidates against COVID-19 in healthy individuals [cited 2021 Apr 30]. https://clinicaltrials.gov/ct2/show/NCT04368728

25. Cochran WG. Sampling techniques. 3rd ed. New York: John Wiley & Sons; 1977.

26. PORDATA. Resident population. Estimates at December 31: total and by age group [cited 2020 Jan 13]. https://www.pordata.pt/en/DB/Municipalities/Search+Environment/Table/5819785

27. Brown LD, Cai TT, DasGupta A. Interval estimation for a binomial proportion. Stat Sci. 2001;16:101–33. https://doi.org/10.1214/ss/1009213286

28. Waller JL, Addy CL, Jackson KL, Garrison CZ. Confidence intervals for weighted proportions. Stat Med. 1994;13:1071–82. PubMed https://doi.org/10.1002/sim.4780131009

29. Ainsworth M, Andersson M, Auckland K, Baillie JK, Barnes E, Beer S, et al.; National SARS-CoV-2 Serology Assay Evaluation Group. Performance characteristics of five immunoassays for SARS-CoV-2: a head-to-head benchmark comparison. Lancet Infect Dis. 2020;20:1390–400. PubMed https://doi.org/10.1016/S1473-3099(20)30634-4

30. Sempos CT, Tian L. Adjusting coronavirus prevalence estimates for laboratory test kit error. Am J Epidemiol. 2021;190:109–15. PubMed https://doi.org/10.1093/aje/kwaa174

31. Rogan WJ, Gladen B. Estimating prevalence from the results of a screening test. Am J Epidemiol. 1978;107:71–6. PubMed https://doi.org/10.1093/oxfordjournals.aje.a112510

32. Bar-On YM, Flamholz A, Phillips R, Milo R. SARS-CoV-2 (COVID-19) by the numbers. eLife. 2020;9:e57309. PubMed https://doi.org/10.7554/eLife.57309

33. Long QX, Liu BZ, Deng HJ, Wu GC, Deng K, Chen YK, et al. Antibody responses to SARS-CoV-2 in patients with COVID-19. Nat Med. 2020;26:845–8. PubMed https://doi.org/10.1038/s41591-020-0897-1

34. Lou B, Li TD, Zheng SF, Su YY, Li ZY, Liu W, et al. Serology characteristics of SARS-CoV-2 infection after exposure and post-symptom onset. Eur Respir J. 2020;56:2000763. PubMed https://doi.org/10.1183/13993003.00763-2020

35. Linton NM, Kobayashi T, Yang Y, Hayashi K, Akhmetzhanov AR, Jung SM, et al. Incubation period and other epidemiological characteristics of 2019 novel coronavirus infections with right truncation: a statistical analysis of publicly available case data. J Clin Med. 2020;9:E538. PubMed https://doi.org/10.3390/jcm9020538

36. Wilson N, Kvalsvig A, Barnard LT, Baker MG. Case-fatality risk estimates for COVID-19 calculated by using a lag time for fatality. Emerg Infect Dis. 2020;26:1339–441. PubMed https://doi.org/10.3201/eid2606.200320

37. Mizumoto K, Chowell G. Estimating risk for death from coronavirus disease, China, January–February 2020. Emerg Infect Dis. 2020;26:1251–6. PubMed https://doi.org/10.3201/eid2606.200233

38. Brazeau N. Verity R. Jenks S. Fu H. Whittaker C. Winskill P. et al. Report 34: COVID-19 infection fatality ratio: estimates from seroprevalence: Imperial College London; October29, 2020 [cited 2021 Aug 11]. https://spiral.imperial.ac.uk/handle/10044/1/83545

39. Lumley T. Analysis of complex survey samples. J Stat Softw. 2004;9. https://doi.org/10.18637/jss.v009.i08

40. Viner RM, Mytton OT, Bonell C, Melendez-Torres GJ, Ward J, Hudson L, et al. Susceptibility to SARS-CoV-2 infection among children and adolescents compared with adults: a systematic review and meta-analysis. JAMA Pediatr. 2021;175:143–56. PubMed https://doi.org/10.1001/jamapediatrics.2020.4573

41. Hobbs CV, Drobeniuc J, Kittle T, Williams J, Byers P, Satheshkumar PS, et al.; CDC COVID-19 Response Team. Estimated SARS-CoV-2 seroprevalence among persons aged <18 years—Mississippi, May–September 2020. MMWR Morb Mortal Wkly Rep. 2021;70:312–5. PubMed https://doi.org/10.15585/mmwr.mm7009a4

42. PORDATA. Resident population. annual average: total and by sex [cited 2020 Jan 23]. https://www.pordata.pt/en/Portugal/Resident+population++annual+average+total+and+by+sex-6

43. Simons D, Shahab L, Brown J, Perski O. The association of smoking status with SARS-CoV-2 infection. hospitalization and mortality from COVID-19: a living rapid evidence review with Bayesian meta-analyses (version 7). Addiction. 2020. PubMed https://doi.org/10.1111/add.15276

44. Paleiron N, Mayet A, Marbac V, Perisse A, Barazzutti H, Brocq FX, et al. Impact of tobacco smoking on the risk of COVID-19: a large scale retrospective cohort study. Nicotine Tob Res. 2021;23:1398–404. PubMed https://doi.org/10.1093/ntr/ntab004

45. Hopkinson NS, Rossi N, El-Sayed Moustafa J, Laverty AA, Quint JK, Freidin M, et al. Current smoking and COVID-19 risk: results from a population symptom app in over 2.4 million people. Thorax. 2021;76:714–22. PubMed https://doi.org/10.1136/thoraxjnl-2020-216422

46. Bar-Zeev Y. Commentary on Simons et al. (2020): public health implications of the suggested association between nicotine. smoking and infection with SARS-CoV-2. Addiction. 2020. PubMed

47. van Westen-Lagerweij NA, Meijer E, Meeuwsen EG, Chavannes NH, Willemsen MC, Croes EA. Are smokers protected against SARS-CoV-2 infection (COVID-19)? The origins of the myth. NPJ Prim Care Respir Med. 2021;31:10. PubMed https://doi.org/10.1038/s41533-021-00223-1

48. Curtis N, Sparrow A, Ghebreyesus TA, Netea MG. Considering BCG vaccination to reduce the impact of COVID-19. Lancet. 2020;395:1545–6. PubMed https://doi.org/10.1016/S0140-6736(20)31025-4

49. US National Library of Medicine. BCG Vaccination to Protect Healthcare Workers Against COVID-19 (BRACE) [cited 2021 Jan 31]. https://clinicaltrials.gov/ct2/show/NCT04327206

50. Rivas MN, Ebinger JE, Wu M, Sun N, Braun J, Sobhani K, et al. BCG vaccination history associates with decreased SARS-CoV-2 seroprevalence across a diverse cohort of health care workers. J Clin Invest. 2021;131:145157. PubMed https://doi.org/10.1172/JCI145157

51. Shioda K, Lau MSY, Kraay ANM, Nelson KN, Siegler AJ, Sullivan PS, et al. Estimating the cumulative incidence of SARS-CoV-2 infection and the infection fatality ratio in light of waning antibodies. Epidemiology. 2021;32:518–24. PubMed https://doi.org/10.1097/EDE.0000000000001361

52. Choe PG, Kang CK, Suh HJ, Jung J, Song KH, Bang JH, et al. Waning antibody responses in asymptomatic and symptomatic SARS-CoV-2 infection. Emerg Infect Dis. 2021;27:327–9. PubMed https://doi.org/10.3201/eid2701.203515

53. Self WH, Tenforde MW, Stubblefield WB, Feldstein LR, Steingrub JS, Shapiro NI, et al.; CDC COVID-19 Response Team; IVY Network. Seroprevalence of SARS-CoV-2 among frontline health care personnel in a multistate hospital network—13 academic medical centers, April–June 2020. MMWR Morb Mortal Wkly Rep. 2020;69:1221–6. PubMed https://doi.org/10.15585/mmwr.mm6935e2

54. Choe PG, Kim KH, Kang CK, Suh HJ, Kang E, Lee SY, et al. Antibody responses 8 months after asymptomatic or mild SARS-CoV-2 infection. Emerg Infect Dis. 2021;27:928–31. PubMed https://doi.org/10.3201/eid2703.204543

55. Dan JM, Mateus J, Kato Y, Hastie KM, Yu ED, Faliti CE, et al. Immunological memory to SARS-CoV-2 assessed for up to 8 months after infection. Science. 2021;371:eabf4063. PubMed https://doi.org/10.1126/science.abf4063

56. Hartley GE, Edwards ES, Aui PM, Varese N, Stojanovic S, McMahon J, et al. Rapid generation of durable B cell memory to SARS-CoV-2 spike and nucleocapsid proteins in COVID-19 and convalescence. Sci Immunol. 2020;5:eabf8891. PubMed https://doi.org/10.1126/sciimmunol.abf8891

57. Ripperger TJ, Uhrlaub JL, Watanabe M, Wong R, Castaneda Y, Pizzato HA, et al. Orthogonal SARS-CoV-2 serological assays enable surveillance of low-prevalence communities and reveal durable humoral immunity. Immunity. 2020;53:925–933.e4. PubMed https://doi.org/10.1016/j.immuni.2020.10.004

58. Wajnberg A, Amanat F, Firpo A, Altman DR, Bailey MJ, Mansour M, et al. Robust neutralizing antibodies to SARS-CoV-2 infection persist for months. Science. 2020;370:1227–30. PubMed https://doi.org/10.1126/science.abd7728

Appendix Table 1. Estimated sample size by stratum for the study in Portugal

Population density	<18 y	18–54 y	≥55 y	Total
Low (<60/km^2)	341	995	991	2,327
Medium (60–500/km^2)	889	2,112	1,504	4,505
High (>500/km^2)	963	2,403	1,796	5,162
Total	2,193	5,510	4,291	11,994

Appendix Table 2. Sample distribution by county (Portugal), population density and age group, each cell represents the number of persons to be sampled in that stratum by county.

Population density	<18 y	18–54 y	≥55 y	Total/county	No. counties	Total
Low	17	50	50	117	20	2,340
Medium	15	35	25	75	60	4,500
High	40	100	75	215	24	5,160
Total					104	12,000

Appendix Table 3. Population weights by stratum after post-stratifying also by sex

Population density		<18 y	18–54 y	≥55 y	Total
Low (<60/km^2)	Men	1.0%	2.9%	2.5%	
	Women	1.0%	2.9%	3.2%	
		2.0%	5.8%	5.7%	13.5%
Medium (60 to	Men	3.6%	9.3%	6.2%	
500/km^2)	Women	3.5%	9.8%	7.8%	
		7.1%	19.0%	14.0%	40.1%
High (>500/km^2)	Men	4.5%	10.4%	6.9%	
	Women	4,3%	11.3%	9.1%	
		8.8%	21.6%	16.0%	46.4%
Total		17.9%	46.4%	35.7%	100.0%

Appendix Table 4. Final sample sizes for the study in Portugal by stratum

Population density	<18 y	18–54 y	≥55 y	Total
Low (<60/km^2)	304	1,017	977	2,298
Medium (60 to 500/km^2)	848	2,461	1,697	5,006
High (>500/km^2)	956	3,017	2,121	6,094
Total	2,108	6,495	4,795	13,398

Appendix Table 5. Sample and Portuguese population statistics for key variables

Characteristic	In sample	In Portuguese population*
Sex		
M	44.7%	47.2%
F	55.3%	52.8%
Age categories		
<18 y	15.7%	17.9%
18–54 y	48.5%	46.4%
≥55 y	35.8%	35.7%
Household size		
1 person	8.5%	21.4%
2 to 4 persons	83.4%	69.1%
≥5 persons	8.0%	9.5%
Education		
Less than high school	31.0%	60.1%
High school, post high school (no undergraduate degree)	25.2%	20.4%
Undergraduate or graduate degree	41.8%	19.5%
Occupation		
Employed	56.6%	52.0%
Unemployed	5.0%	4.5%
Student	18.6%	19.1%
Retired	14.5%	19.6%
Professional sector		
Commerce	8.0%	13.9%
Industry	7.2%	17.3%
Building	2.5%	6.2%
Administration/services	25.9%	28.6%
Education	18.1%	8.7%
Health	11.7%	2.6%
Transportation	2.8%	4.4%
Other	23.8%	18.3%

*Source: INE – Statistics Portugal.

Appendix Table 6. Sociodemographic characteristics of participants

Characteristic	Total (n = 13 398)
Sex. n (%)	
M	5,985 (44.7%)
F	7,413 (55.3%)
Age (years)	
Mean (standard deviation)	43.3 (18.8)
Minimum	1.0
Maximum	92.0
Age categories. n (%)	
<18 y	2,108 (15.7%)
18–54 y	6,495 (48.5%)
≥55 y	4,795 (35.8%)
Age <18 y (years)	
Mean (standard deviation)	12.7 (3.8)
Minimum	1
Maximum	17
Age 18–54 y (years)	
Mean (standard deviation)	38.7 (9.2)
Minimum	18
Maximum	54
Age ≥55 y (years)	
Mean (standard deviation)	62.0 (6.5)
Minimum	55
Maximum	92
Population density. n (%)	
Low	2,298 (17.2%)
Medium	5,006 (37.4%)
High	6,094 (45.5%)
Household size. n (%)	
1 person	1,141 (8.5%)
2 to 4 persons	11,139 (83.4%)
≥5 persons	1,069 (8.0%)
Education. n (%)	
Less than high school	4,145 (31.0%)
High school. post high school (no undergraduate degree)	3,373 (25.2%)
Undergraduate or graduate degree	5,603 (41.8%)
Other	270 (2.0%)
Occupation. n (%)	
Employed	7,584 (56.6%)
Unemployed	668 (5.0%)
Student	2,488 (18.6%)
Retired	1,943 (14.5%)
Disability	143 (1.1%)
House worker	223 (1.7%)
Other	333 (2.5%)
Professional sector. n (%)	
Commerce	594 (8.0%)
Industry	540 (7.2%)
Building	190 (2.5%)
Administration/services	1,930 (25.9%)
Education	1,351 (18.1%)
Health	875 (11.7%)
Transportation	207 (2.8%)
Other	1,772 (23.8%)
For employed workers	
Current working arrangement. n (%)	
Teleworking	
No	6,480 (85.4%)
Yes	1,104 (14.6%)
Physically at work. contact with colleagues	
No	263 (13.3%)
Yes	6,579 (86.7%)
Physically at work. contact with public	
No	4,184 (55.2%)
Yes	3,400 (44.8%)

Appendix Table 7. Health and clinical characteristics of participants

Characteristic	Total (n = 13,398)
Body Mass Index* (kg/m^2)	
Mean	25.9
Standard deviation	4.4
Body Mass Index* n (%)	
Underweight (<18.50 kg/m^2)	187 (1.7%)
Normal (18.50 – 24.99 kg/m^2)	5,165 (45.8%)
Overweight (25.00 – 29.99 kg/m^2)	4,166 (36.9%)
Obese (≥30.00 kg/m^2)	1,766 (15.7%)
Smoking status. n (%)	
Non-smoker	9,235 (68.9%)
Ex-smoker	2,298 (17.2%)
Smoker	1,862 (13.9%)
<20 cigarettes/day	1,689 (90.7%)
≥20 cigarettes/day	173 (9.3%)
Physical exercise (3x/week for at least 30 min.). n (%)	
No	7,590 (56.7%)
Yes	5,808 (43.3%)
Influenza vaccine in the last year. n (%)	
No	10,722 (80.0%)
Yes	2,676 (20.0%)
BCG vaccine. n (%)	
No	688 (5.1%)
Yes	10,672 (79.7%)
Don't know	2,038 (15.2%)
Chronic disease† n (%)	3,717 (27.7%)
Diabetes mellitus	467 (12.6%)
Renal insufficiency with hemodialysis	5 (0.1%)
Chronic obstructive pulmonary disease (COPD)	148 (4.0%)
Asthma	701 (18.9%)
Hypertension	1,189 (32.0%)
Oncologic disease	252 (6.8%)
Cardiovascular disease	368 (9.9%)
Autoimmune disease	536 (14.4%)
Hepatic disease	39 (1.0%)
Illness with immune suppression treatment	52 (1.4%)
Other	757 (20.4%)
Number of chronic diseases. n (%)	
0	10,382 (77.5%)
1–2	2,890 (21.6%)
≥3	126 (0.9%)

*BMI calculated only for adults (>18 y-old);
†Participants could choose more than one chronic disease.

Appendix Table 8. Asymptomatic and pauci-symptomatic infections by population density and age group. adjusted for sensitivity and specificity*

Characteristic	No. participants	Asymptomatic (95% CI)	Pauci-symptomatic (95% CI)*
Population density			
Low	33	21.4% (13.1- 37.8)	26.9% (16.7- 43.3)
Medium	85	23.4% (16.2; 33.9)	25.1% (17.3- 35.6)
High	178	13.9% (9.8- 20.7)	16.3% (11.6- 23.2)
Age group			
<18 y	49	37.2% (24.2- 49.6)	39.6% (28.2- 52.0)
18–54 y	152	13.9% (9.7- 21.4)	14.3% (10.0- 21.9)
≥55 y	95	11.0% (6.9- 21.0)	16.6% (10.8- 26.9)
Overall	296	17.4% (14.1- 22.9)	19.9% (16.1- 25.4)

*Seropositive persons with less than three symptoms and without sudden loss of smell or taste.

Appendix Table 9. Sample distribution of seropositive and non-seropositive by sociodemographic characteristics

Characteristic	Seropositive (n = 296)	Non-seropositive (n = 13,102)	p value
Sex			
M	137 (46.3%)	5,848 (44.6%)	0.572
F	159 (53.7%)	7,254 (55.4%)	
Age (years)			
<18	49 (16.6%)	2,059 (15.7%)	0.407
18–54	152 (51.4%)	6,343 (48.4%)	
≥55	95 (32.1%)	4,700 (35.9%)	
Household size			
1 person	31 (10.5%)	1,110 (8.5%)	0.347
2 to 4 persons	238 (80.4%)	10,901 (83.5%)	
≥5 persons	27 (9.1%)	1,042 (8%)	
Education			
Less than high school	84 (28.4%)	4,061 (31%)	0.397
High school. post high school (no undergraduate degree)	81 (27.4%)	3,292 (25.1%)	
Undergraduate or graduate degree	121 (40.9%)	5,482 (41.9%)	
Other	10 (3.4%)	260 (2%)	
Occupation			
Employed	174 (58.8%)	7,410 (56.6%)	0.106
Unemployed	17 (5.7%)	651 (5%)	
Student	55 (18.6%)	2,433 (18.6%)	
Retired	32 (10.8%)	1,911 (14.6%)	
Disability	1 (0.3%)	142 (1.1%)	
House worker	3 (1%)	220 (1.7%)	
Other	14 (4.7%)	319 (2.4%)	
Professional sector, n (%)			
Commerce/industry/building	35 (20.2%)	1,289 (17.7%)	0.144
Administration/services	50 (28.9%)	1,880 (25.8%)	
Education	22 (12.7%)	1,329 (18.2%)	
Health	27 (15.6%)	848 (11.6%)	
Transportation	7 (4%)	200 (2.7%)	
Other	32 (18.5%)	1,751 (24%)	
Employed workers			
Current working arrangements			
Teleworking			
No	155 (89.1%)	6,325 (85.4%)	0.169
Yes	19 (10.9%)	1,085 (14.6%)	
Physically at work: contact with colleagues			
No	7 (4.4%)	256 (3.8%)	0.711
Yes	152 (95.6%)	6,427 (96.2%)	
Physically at work: contact with the public			
No	100 (57.5%)	4,084 (55.1%)	0.537
Yes	74 (42.5%)	3,326 (44.9%)	

Appendix Table 10. Sample distribution of seropositive and non-seropositive by health and clinical characteristic

Characteristic	Seropositive (n = 296)	Non-swropositive (n = 13,102)	p value
Body mass index*			
Underweight (<18.50 kg/m^2)	0 (0%)	187 (1.7%)	0.104
Normal weight (18.50–24.99 kg/m^2)	100 (40.5%)	5,033 (45.6%)	
Overweight (25.00–29.99 kg/m^2)	104 (42.1%)	4,098 (37.1%)	
Obese (≥30.00 kg/m^2)	43 (17.4%)	1,723 (15.6%)	
Smoking status. n (%)			
Non-smoker	223 (75.3%)	9,012 (68.8%)	0.007
Ex-smoker	50 (16.9%)	2,248 (17.2%)	
Smoker	23 (7.8%)	1,839 (14%)	
<20 cigarettes/day	23 (100%)	1,666 (90.6%)	0.122
≥20 cigarettes/day	0 (0.0%)	173 (9.4%)	
Physical exercise			
No	174 (58.8%)	7,416 (56.6%)	0.454
Yes	122 (41.2%)	5,686 (43.4%)	
Influenza vaccine in the last year			
No	234 (79.1%)	10,488 (80%)	0.672
Yes	62 (20.9%)	2,614 (20%)	
BCG vaccine			
No	17 (5.7%)	671 (5.1%)	0.571
Yes	240 (81.1%)	10,432 (79.6%)	
Do not know	39 (13.2%)	1,999 (15.3%)	

Characteristic	Seropositive (n = 296)	Non-swropositive (n = 13,102)	p value
BCG vaccine			
No	17 (6.6%)	671 (6%)	0.704
Yes	240 (93.4%)	10,432 (94%)	
Chronic disease			
No	222 (75%)	9,459 (72.2%)	0.286
Yes	74 (25%)	3,643 (27.8%)	
If yes			
Diabetes mellitus			
No	66 (89.2%)	3,184 (87.4%)	0.646
Yes	8 (10.8%)	459 (12.6%)	
Renal insufficiency with hemodialysis			
No	74 (100%)	3,638 (99.9%)	>0.9999
Yes	0 (0.0%)	5 (0.1%)	
Chronic obstructive pulmonary disease (COPD)			
No	72 (97.3%)	3,497 (96%)	>0.9999
Yes	2 (2.7%)	146 (4%)	
Asthma			
No	58 (78.4%)	2,958 (81.2%)	0.539
Yes	16 (21.6%)	685 (18.8%)	
Hypertension			
No	53 (71.6%)	2,475 (67.9%)	0.501
Yes	21 (28.4%)	1,168 (32.1%)	
Oncologic illness			
No	68 (91.9%)	3,397 (93.2%)	0.646
Yes	6 (8.1%)	246 (6.8%)	
Cardiovascular disease			
No	68 (91.9%)	3,281 (90.1%)	0.602
Yes	6 (8.1%)	362 (9.9%)	
Autoimmune disease			
No	61 (82.4%)	3,120 (85.6%)	0.436
Yes	13 (17.6%)	523 (14.4%)	
Hepatic disease			
No	74 (100%)	3,604 (98.9%)	>0.9999
Yes	0 (0.0%)	39 (1.1%)	
Immunosuppression treatment			
No	73 (98.6%)	3,592 (98.6%)	0.972
Yes	1 (1.4%)	51 (1.4%)	
Other chronic disease			
No	60 (81.1%)	2,900 (79.6%)	0.755
Yes	14 (18.9%)	743 (20.4%)	
No. chronic diseases†			
0	222 (75%)	9,459 (72.2%)	0.532
1–2	72 (24.3%)	3,515 (26.8%)	
≥3	2 (0.7%)	128 (1%)	

*BMI calculated only for adults (>18 y-old).
†Participants could choose more than one chronic disease.

Appendix Table 11. Sample distribution of seropositive and non-seropositive by epidemiologic characteristics

Characteristic	Seropositive (n = 296)	Non-seropositive (n = 13,102)	p value
Were you in contact with someone infected? n (%)			
No	27 (9.1%)	3,594 (27.4%)	<0.0001
Yes	155 (52.4%)	870 (6.6%)	
Do not know	114 (38.5%)	8,638 (65.9%)	
Where was this potential contact? n (%)			
Household	69 (44.5%)	226 (26%)	<0.0001
Work	46 (29.7%)	386 (44.4%)	
Family outsider household	28 (18.1%)	182 (20.9%)	
Healthcare institution	3 (1.9%)	45 (5.2%)	
Do not know	9 (5.8%)	31 (3.6%)	
Was someone in your household diagnosed with COVID-19? n (%)			
No	181 (61.1%)	12,816 (97.8%)	<0.0001
Yes	115 (38.9%)	286 (2.2%)	
Were you diagnosed as a suspected COVID-19 case?			
No	148 (50%)	1,2581 (96%)	<0.0001
Yes	148 (50%)	521 (4%)	
If you had a SARS-CoV-2 test, what was the result?			
Positive	112 (66.3%)	24 (1.2%)	<0.0001
Negative	49 (29%)	1,982 (97.9%)	
Inconclusive	8 (4.7%)	19 (0.9%)	
If you had an antibody test before, what was the result?			
Positive	21 (77.8%)	10 (4.3%)	<0.0001
Negative	5 (18.5%)	214 (92.2%)	
Inconclusive	1 (3.7%)	8 (3.4%)	

Appendix Table 12. Sample distribution of seropositive and non-seropositive by reported symptoms

Characteristic	Seropositive (n = 296)	Non-seropositive (n = 13 102)	p value
Since the beginning of the pandemic (March 2, 2020), did you have any of the following symptoms. n (%)			
Loss of smell			
No	179 (60.7%)	12,590 (98%)	<0.0001
Yes	116 (39.3%)	256 (2%)	
Loss of taste			
No	170 (57.6%)	12,489 (97.2%)	<0.0001
Yes	125 (42.4%)	357 (2.8%)	
Fever (≥38°C)			
No	198 (67.1%)	12,060 (93.9%)	<0.0001
Yes	97 (32.9%)	786 (6.1%)	
Dry cough			
No	189 (64.1%)	10,725 (83.5%)	<0.0001
Yes	106 (35.9%)	2,121 (16.5%)	
Cough with mucus			
No	246 (83.4%)	11,219 (87.3%)	0.045
Yes	49 (16.6%)	1,627 (12.7%)	
Cough with blood			
No	295 (100%)	12,817 (99.8%)	>0.9999
Yes	0 (0%)	29 (0.2%)	
Muscle or joint pain			
No	150 (50.8%)	9,590 (74.7%)	<0.0001
Yes	145 (49.2%)	3,256 (25.3%)	
Sore throat			
No	219 (74.2%)	10,075 (78.4%)	0.084
Yes	76 (25.8%)	2,771 (21.6%)	
Headaches			
No	146 (49.5%)	8,507 (66.2%)	<0.0001
Yes	149 (50.5%)	4,339 (33.8%)	
General weakness			
No	181 (61.4%)	11,368 (88.5%)	<0.0001
Yes	114 (38.6%)	1,478 (11.5%)	
Respiratory difficulty			
No	244 (82.7%)	11,790 (91.8%)	<0.0001
Yes	51 (17.3%)	1,056 (8.2%)	
Vomiting			
No	278 (94.2%)	12,423 (96.7%)	0.020
Yes	17 (5.8%)	423 (3.3%)	
Diarrhea			
No	202 (68.5%)	10,548 (82.1%)	<0.0001
Yes	93 (31.5%)	2,298 (17.9%)	

Characteristic	Seropositive (n = 296)	Non-seropositive (n = 13 102)	p value
Nausea			
No	264 (89.5%)	11,981 (93.3%)	0.011
Yes	31 (10.5%)	865 (6.7%)	
Chills			
No	224 (75.9%)	11,706 (91.1%)	<0.0001
Yes	71 (24.1%)	1,140 (8.9%)	
Lack of appetite			
No	211 (71.5%)	11,978 (93.2%)	<0.0001
Yes	84 (28.5%)	868 (6.8%)	
Feeling tired			
No	142 (48.1%)	9,303 (72.4%)	<0.0001
Yes	153 (51.9%)	3,543 (27.6%)	
Rashes			
No	279 (94.6%)	12,118 (94.3%)	0.858
Yes	16 (5.4%)	728 (5.7%)	
Rhinorrhea			
No	211 (71.5%)	10,238 (79.7%)	0.001
Yes	84 (28.5%)	2,608 (20.3%)	
Loss of consciousness			
No	293 (99.3%)	12,783 (99.5%)	0.821
Yes	2 (0.7%)	63 (0.5%)	
Total number of symptoms since March 2, 2020			
0	48 (16.2%)	6,516 (49.7%)	<0.0001
1–2	35 (11.8%)	1,780 (13.6%)	
≥3	213 (72%)	4,806 (36.7%)	
Asymptomatic			
No	248 (83.8%)	6,586 (50.3%)	<0.0001
Yes	48 (16.2%)	6,516 (49.7%)	
Pauci-symptomatic			
No	241 (81.4%)	5,751 (43.9%)	<0.0001
Yes	55 (18.6%)	7,351 (56.1%)	

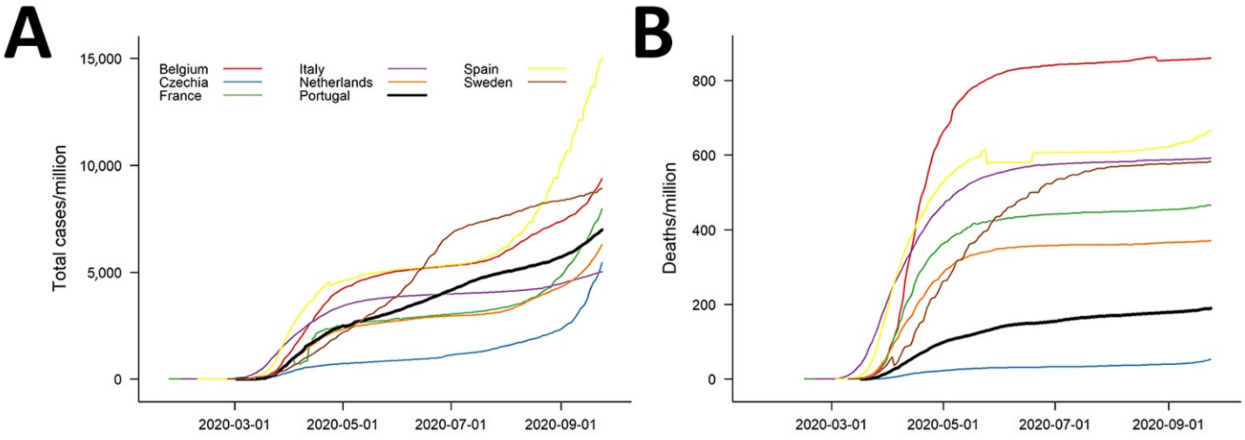

Appendix Figure 1. Cases of and deaths per million persons from coronavirus disease for selected countries in Europe. Numbers of cumulative cases (top) and deaths (bottom) per million persons for countries approximately the same size as Portugal (i.e., ≈10 million inhabitants) in Europe (Belgium, Czechia, Netherlands, and Sweden) and 3 neighboring countries (Spain, the only country that shares a land border with Portugal, France, and Italy). Portugal had a similar number of cases, but a relatively low number of deaths during the first 6 months of the pandemic. Data were obtained from https://ourworldindata.org/coronavirus.

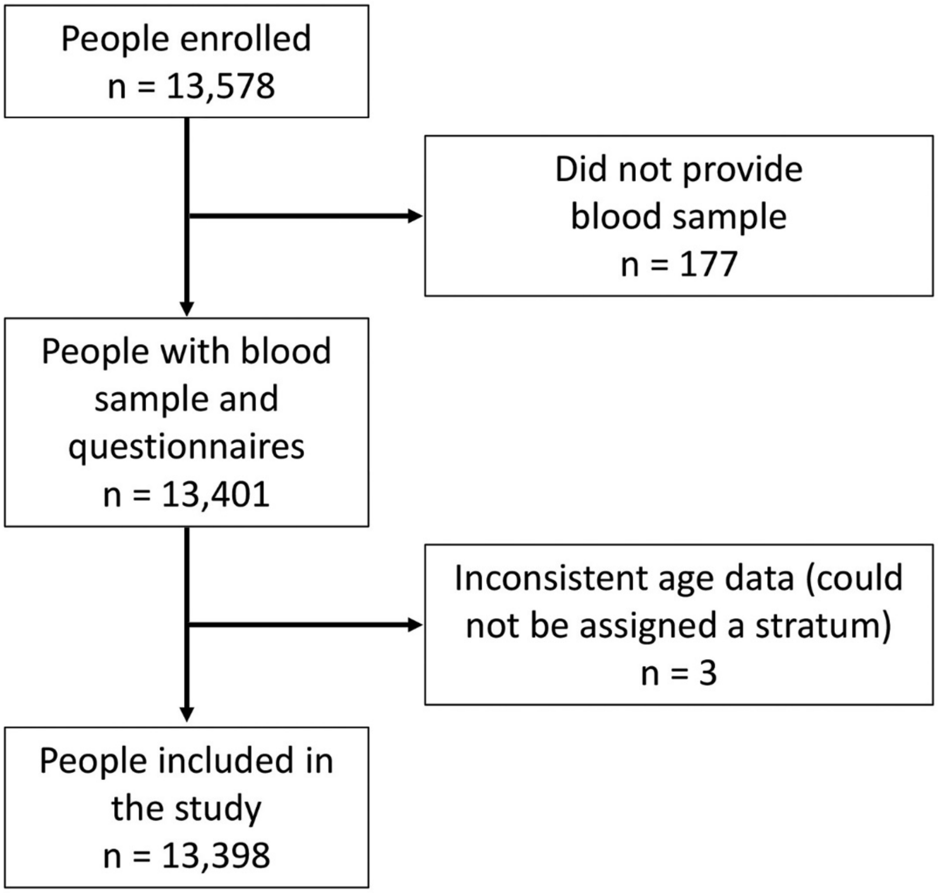

Appendix Figure 2. Flowchart for study participants.

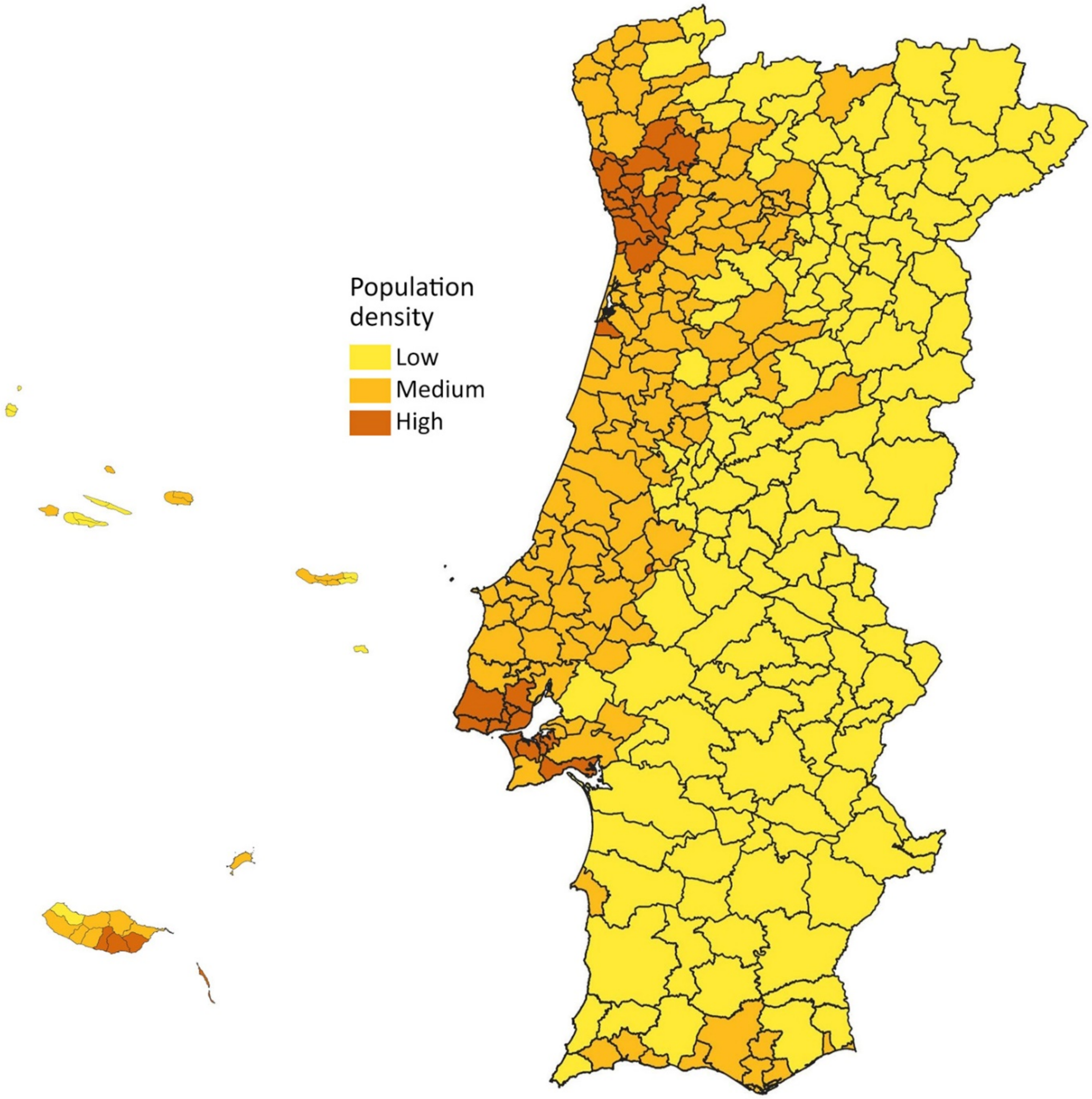

Appendix Figure 3. Seroprevalence of antibodies against SARS-CoV-2 in Portugal by population density. Map of Portugal subdivided by counties, with seroprevalence results for low population density 1.4% (95% CI 1.1%–2.2%), medium population density 1.6% (95% CI 1.4%– 2.1%), and high population density 2.9% (95% CI 2.5%–3.4%). We included in this study 104 counties from the 308 in the whole country, but we extrapolated to all counties based on population density shown (not all colored counties were sampled).

Questionnaire

The participants answered the following questionnaire (the original version is in Portuguese). Most questions were single choice. unless it explicit says “Select all applicable”

Sociodemographic

1) Sex ☐ Male ☐ Female

2) Age ☐☐ years

3) Nationality ☐ Portuguese ☐ Other

4) Place of residence [Dropdown box with counties]

5) Weight ☐☐☐ Kg

6) Height ☐ . ☐☐ m

7) How many people live in your household (including you)? ☐☐ people

8) What is the highest level of schooling that you completed or obtained an equivalency to?

(Use the last level that you completed. If you don’t know what is the best option. choose “Other situation.”)

X Did not go to school

X Completed the 1st, 2nd, or 3rd year of school

X Completed the 4th or 5th year of school

X Completed the 6th, 7th, or 8th year of school

X Completed the 9th, 10th, or 11th year of school

X Completed high school (12th year. or other equivalent degree)

X Completed non-university post-high school degree (professional training)

X Completed an university degree (undergraduate. master. PhD)

X Other situation

9) What is your current professional situation? ☐ Active worker

☐ Volunteer worker

☐ Unemployed

☐ Student

☐ Retired

☐ Disability/Medical leave

☐ Homemaker

☐ Other

9.1.1) If active/volunteer worker. what sector?

☐ Commerce ☐ Security

☐ Industry ☐ Cleaning

☐ Building ☐ Health

☐ Administration and services ☐ Health without clinical intervention

☐ Transportation ☐ Carer of dependent people

☐ Militarized forces ☐ Academics/Education

☐ Other

9.1.2) If active/volunteer worker. what is your current working arrangements? Select all applicable.

☐ Teleworking

☐ Physically at work. no contact with colleagues

☐ Physically at work. with contact with colleagues

☐ Physically at work. with contact with the public

Health priors

10) Smoking habits ☐ Non-smoker

☐ Ex-smoker

☐ Smoker: ☐ <20 cigarettes/day

☐ ≥20 cigarettes/day

11) Do you exercise regularly (3 times/week for at least 30 min.)? ☐ Yes ☐ No

12) In the last year. did you take the flu vaccine? ☐ Yes ☐ No

13) Did you ever get the BCG vaccine (for tuberculosis)? ☐ Yes ☐ No ☐ Don't know

14) Do you have a chronic disease? ☐ Yes ☐ No

15) Do you have any of the following chronic diseases? Select all applicable.

☐ Diabetes mellitus

☐ Renal insufficiency with hemodialysis

☐ Chronic obstructive pulmonary disease (COPD)

☐ Asthma

☐ Arterial hypertension

☐ Oncological disease

☐ Cardiovascular disease

☐ Autoimmune disease

☐ Hepatic disease

☐ Illness with immunosuppression treatment

☐ None of the above

Factors possibly associated with infection by SARS-CoV-2

16) Since the beginning of the pandemic (2 March 2020). did you have any of the following symptoms? What was the severity (1 = mild to 5 = severe)? Select all applicable.

☐ Sudden loss of smell

Severity ☐ 1 ☐ 2 ☐ 3 ☐ 4 ☐ 5

☐ Loss of taste

Severity ☐ 1 ☐ 2 ☐ 3 ☐ 4 ☐ 5

☐ Fever (≥38°C)

Severity ☐ 1 ☐ 2 ☐ 3 ☐ 4 ☐ 5

☐ Dry cough

Severity ☐ 1 ☐ 2 ☐ 3 ☐ 4 ☐ 5

☐ Cough with mucus

Severity ☐ 1 ☐ 2 ☐ 3 ☐ 4 ☐ 5

☐ Cough with blood

Severity ☐ 1 ☐ 2 ☐ 3 ☐ 4 ☐ 5

☐ Muscle or joint pain

Severity ☐ 1 ☐ 2 ☐ 3 ☐ 4 ☐ 5

☐ Sore throat

Severity ☐ 1 ☐ 2 ☐ 3 ☐ 4 ☐ 5

☐ Headache

Severity ☐ 1 ☐ 2 ☐ 3 ☐ 4 ☐ 5

☐ General weakness

Severity ☐ 1 ☐ 2 ☐ 3 ☐ 4 ☐ 5

☐ Respiratory difficulty

Severity ☐ 1 ☐ 2 ☐ 3 ☐ 4 ☐ 5

☐ Vomit

Severity ☐ 1 ☐ 2 ☐ 3 ☐ 4 ☐ 5

☐ Diarrhea

Severity ☐ 1 ☐ 2 ☐ 3 ☐ 4 ☐ 5

☐ Nausea

Severity ☐ 1 ☐ 2 ☐ 3 ☐ 4 ☐ 5

☐ Chills

Severity ☐ 1 ☐ 2 ☐ 3 ☐ 4 ☐ 5

☐ Lack of appetite

Severity ☐ 1 ☐ 2 ☐ 3 ☐ 4 ☐ 5

☐ Tiredness

Severity ☐ 1 ☐ 2 ☐ 3 ☐ 4 ☐ 5

☐ Rashes

Severity ☐ 1 ☐ 2 ☐ 3 ☐ 4 ☐ 5

☐ Runny nose (rhinorrhea)

Severity ☐ 1 ☐ 2 ☐ 3 ☐ 4 ☐ 5

☐ Loss of consciousness

Severity ☐ 1 ☐ 2 ☐ 3 ☐ 4 ☐ 5

16.1.1) When did the first symptoms start? ☐☐ / ☐☐ / ☐☐☐☐

16.1.2.1) Do you still have the symptoms? ☐ Yes ☐ No

16.1.2.2) If not. when did the symptoms end? ☐☐ / ☐☐ / ☐☐☐☐

16.2) Did you have any of the symptoms in the past 15 days? ☐ Yes ☐ No

16.2.1) If yes. which? [Dropdown box multiselect]

17) Did you have any contact with someone infected with SARS-CoV-2?

☐ Yes ☐ No ☐ Don't know

17.1) If yes. when was the probable date of contact with an infected person?

☐☐ / ☐☐ / ☐☐☐☐

17.2) Where was the probable contact with an infected person?

☐ Home

☐ Work

☐ Family outsider the household

☐ Health institution

☐ Don't know

18) Was anyone in your household diagnosed with COVID-19? ☐ Yes ☐ No

18.1) If yes. what was the date of the diagnosis? ☐☐ / ☐☐ / ☐☐☐☐

19) Were you at any moment diagnosed with COVID-19? ☐ Yes ☐ No

19.1) If yes. what was the date of the diagnosis? ☐☐ / ☐☐ / ☐☐☐☐

19.2) Who diagnosed you?

☐ SNS24

☐ Private hospital

☐ Public hospital

☐ Private doctor

20) Before this study did you take a COVID-19 test? ☐ Yes ☐ No

20.1) If yes. what type of test?

☐ Nose swab

☐ Blood draw

☐ Finger prick

☐ Other

☐ Don't know

20.2) When did you take the test? ☐☐ / ☐☐ / ☐☐☐☐

20.3) Where did you take the test?

☐ Private hospital

☐ Public hospital

☐ Private laboratory

☐ At home

20.4) What was the result of the test?

☐ Positive

☐ Negative

☐ Inconclusive

21) Were you hospitalized due to COVID-19? ☐ Yes ☐ No

21.1) Date of hospitalization ☐☐ / ☐☐ / ☐☐☐☐

21.2) Date of discharge ☐☐ / ☐☐ / ☐☐☐☐

22) Are you cured? ☐ Yes ☐ No ☐ Don't know

22.1) Who said you were cured?

☐ SNS24

☐ Private hospital

☐ Public hospital

☐ Private doctor

22.2) Did you take a test to confirm cure? ☐ Yes ☐ No

22.3) Date when you were considered cured ☐☐ / ☐☐ / ☐☐☐☐

23) Before this study did you take an immunity test for COVID-19? ☐ Yes ☐ No

23.1) When did you take the test? ☐☐ / ☐☐ / ☐☐☐☐

23.2) Where did you take the test?

☐ Home

☐ Clinic

☐ Hospital

☐ Pharmacy

☐ Private laboratory

23.3) What was the result of the test?

☐ Positive

☐ Negative

☐ Inconclusive

Submit

Correcting the Asymptomatic and Pauci-Symptomatic Prevalence Estimates with Test Sensitivity and Specificity

The proportion of asymptomatic observed in our weighted sample was adjusted taking into consideration the sensitivity and specificity of the test, using the following formula

$$A_{adj} = \frac{AP_m - (1 - S_p)A_S}{P_m + S_P - 1}.$$

where A is the observed weighted proportion of asymptomatic in the seropositive participants P_m is the measured seroprevalence, A_S is the observed proportion of asymptomatic in the full sample, Sp is the test specificity, and A_{adj} is the final adjusted proportion of asymptomatic.

Derivation of the Formula Based on Conditional Probabilities and Bayes' Law

Consider these events/statements

Ab, having antibodies

T^+, having a positive antibody test, and the corresponding probability $P_m = P[T^+]$

T^-, $\approx T^+$ (having a negative antibody test)

Asym, being asymptomatic, and the corresponding probability A_s = P[Asym]

And consider the following notations for the conditional probabilities:

S = P[T^+| Ab], probability of positive test result and having antibodies.

S_p = P[T^-|~Ab], probability of negative test result and not having antibodies.

A = P[Asym | T^+], probability of being asymptomatic and having a positive test result

A_{adj} = P[Asym | Ab], probability of being asymptomatic and having antibodies.

Taking into account that

P[T^+| Asym] = P[Ab | Asym]×S + (1- P[Ab |Asym])×(1-S_p)

We first obtain (1)

$$\mathrm{P[Ab \mid Asym]} = \frac{\mathrm{P[T^+ \mid Asym]} + \mathrm{Sp} - 1}{\mathrm{S} + \mathrm{Sp} - 1}$$

Now. our quantity of interest can be calculated as (2)

$$\mathrm{P[Asym \mid Ab]} = \frac{\mathrm{P[Ab \mid Asym]} \times \mathrm{P[Asym]}}{\mathrm{P[Ab]}}$$

and we also have (3)

$$\mathrm{P[T^+ \mid Asym]} = \frac{\mathrm{P[\,Asym \mid T^+]} \times \mathrm{P[T^+]}}{\mathrm{P[Asym]}}$$

Thus, replacing (3) in (1), (1) in (2), and using the Rogan–Gladen formula to calculate P[Ab] via P[T^+], S and S_p (shown in Methods above) we obtain the desired result.
